# Rapid Cold Hardening Confers a Transient Increase in Low Temperature Survival in Diapausing *Chilo suppressalis* Larvae

**DOI:** 10.3390/insects9020053

**Published:** 2018-05-09

**Authors:** Guangping Yang, Jihui Wen, Yongqiang Han, Maolin Hou

**Affiliations:** 1 State Key Laboratory for Biology of Plant Diseases and Insect Pests, Institute of Plant Protection, Chinese Academy of Agricultural Sciences, Beijing 100193, China; 2009newlife@sina.com; 2 Institute of Plant Protection, Hunan Academy of Agricultural Sciences, Changsha 410128, China; jihuiwen2005@126.com (J.W.); hanyongqiang1984@163.com (Y.H.); 3 Southern Regional Collaborative Innovation Center for Grain and Oil Crops in China, Changsha 410124, China; 4 Scientific Observing and Experimental Station of Crop Pests in Guilin, Ministry of Agriculture, Guilin 541399, China

**Keywords:** striped stem borer, rapid cold hardening, cold tolerance, overwintering, diapause

## Abstract

The striped stem borer, *Chilo suppressalis* (Walker), overwinters as a diapausing larva. The diapausing larvae were tested for a rapid cold hardening (RCH) response and its role in the insect’s survival of sub-zero temperatures. When laboratory-reared diapausing larvae were transferred directly from the rearing temperature of 25 °C to −14 °C and maintained there for 2 h, 21% survived. Acclimation of diapausing larvae for 4 h at 5 °C before their exposure for 2 h to −14 °C increased survival to approximately 41%, indicating an RCH response. Durability of RCH effects on low temperature survival was less than 1 h. Although transient in the test, the increased survival acquired through rapid cold hardening may play a role in preparing the diapausing larvae for abrupt temperature drops in the field that would otherwise be lethal.

## 1. Introduction

Cold tolerance is important in defining an insect’s distribution and survival [1]. Cold tolerance can be achieved through long-term cold acclimatization, where the overwintering insects experience natural and gradual temperature changes; and through rapid cold hardening (RCH), where the overwintering insects experience a natural sudden temperature decrease for a short duration [2]. In natural environments, overwintering insects may be exposed to sudden decreases in temperature in spring and autumn when seasonal adaptations are either incomplete or receding [3,4]. In laboratory tests, when pre-treated by a non-lethal low temperature, insects are able to survive a subsequent cold event that would otherwise be lethal, which is an indication of RCH [4]. While seasonal acclimatization may take a long time to increase low temperature survival, RCH can enhance cold tolerance in such a short time as 30 min [5,6]. RCH has been documented across numerous insect taxa to enhance survival to acute cold stress [4,5,6,7,8,9,10,11,12,13,14], however the cold tolerance gained through RCH is transient [3,8,9,10]. 

The striped stem borer, *Chilo suppressalis* (Walker) (Lepidoptera: Crambidae), is an insect pest of rice and is distributed in all the rice planting areas in China (from 18° N to 46° N) [15]. The insect enters diapause in autumn principally in response to a short day length [16] and overwinters as diapausing larvae in rice stubble near the soil [17]. The overwintering *C. suppressalis* larvae avoid freezing by supercooling, both progressively decreasing temperatures and diapause contribute to supercooling capacity in the overwintering larvae [18]. Decreasing soil moisture also adds to their cold tolerance, with supercooling capacity at the highest when the saturated soil water content is 25% [19]. The supercooling capacity is the lowest in the winter and lower in the over-wintering population in water-oat than in rice [15]. Both diapause and cold-acclimation are essential for the avoidance of freezing injury due to their roles in accumulating glycerol and activating aquaporin [18]. Studies on cold tolerance in diapausing *C. suppressalis* larvae have focused on seasonal acclimatization. However, the overwintering insects may also experience sudden temperature decreases in the autumn and early winter. Qiang, C.-K., et al. [10] detected RCH in non-diapausing *C. suppressalis* mature larvae. It is not clear if RCH also occurs and contributes to low temperature survival in the diapausing/overwintering *C. suppressalis* larvae.

The present study aims to determine if RCH occurs in diapausing *C. suppressalis* larvae and contributes to survival of the larvae at sub-zero temperatures during overwintering and how long the effects of RCH on low temperature survival can last.

## 2. Materials and Methods

### 2.1. Insects and Diapause Induction

Newly hatched *C. suppressalis* larvae from a laboratory culture maintained for 5 years were reared in groups at 10 neonates per glass tube (diameter 2.5 cm, length 10 cm) on an artificial diet [20] in a climate chamber RXZ-260B (Jiangnan Instrument Plant, Ningbo, China) at 25 ± 1 °C, 75 ± 5% relative humidity and LD12:12. From the third stadium, the larvae were reared individually in glass tubes and the diet was replenished one time at 10 d after individual rearing was initiated. It is not possible to distinguish diapausing larvae from non-diapausing larvae from their appearance in this insect species. *C. suppressalis* larvae that did not pupate within 51 d under the mentioned conditions were considered to have entered diapause [16].

### 2.2. Determination of Discriminating Temperature

In the induction and detection of RCH, a standard cold shock temperature (discriminating temperature) has to be established for comparison of survival at low temperatures. The discriminating temperature is defined as the low temperature that resulted in 80% mortality [4,8]. The diapausing larvae were exposed directly from the rearing temperature of 25 °C [4] to a series of sub-zero temperatures (−11, −12, −13, −14, and −15 °C) for an exposure period of 2 h using controlled climate chambers VM04/100 (Heraeus Votsch, Hanau, Germany). The sub-zero temperatures and the treatment duration were employed so that mortalities of the larvae would approximate 80% according to a pilot study. After the treatment, the larvae were re-warmed at 1 °C/min to the rearing temperature (25 °C) and then the larvae were individually transferred to the artificial diet in glass tubes. Survival was assessed at 24 h after the sub-zero temperature treatment. The larvae were recorded as dead if they showed no movement when touched with a soft brush. In a random design, 10 groups of 15 diapausing larvae were tested for each of the sub-zero temperatures. 

### 2.3. Induction and Detection of RCH Response

To detect if RCH occurs in diapausing *C. suppressalis* larvae and the conditions for its occurrence, 15 randomly selected larvae were individually transferred from the rearing temperature (25 °C) to 5 °C, which are employed in previous studies [9], in a glass tube and maintained there for 1, 2, 3, or 4 h. Then, the larvae were immediately subjected to the established discriminating temperature of −14 °C for 2 h in the temperature chamber. Thereafter, the larvae were re-warmed at 1 °C/min to the rearing temperature. Survival was assessed as above. The observation was repeated 10 times for each of the 5 °C steps.

### 2.4. Extent of RCH Response

To assess the range of temperatures where RCH increases survival of diapausing *C. suppressalis* larvae, survival of larvae with and without RCH was compared at a series of sub-zero temperatures. Diapausing larvae that had been subjected to RCH (at 5 °C for 4 h) were transferred to temperatures at and around the discriminating temperature (i.e., −12, −13, −14, −15, and −16 °C) and maintained there for 2 h. Larvae directly transferred from the rearing temperature to the sub-zero temperatures were used as the control. Then, the temperatures were increased to the rearing temperature at 1 °C/min and survival of the larvae was checked as above. For each of the sub-zero temperatures, 10 random replicates of 15 larvae were tested.

### 2.5. Durability of RCH Response

To determine the durability of RCH response, fifteen diapausing *C. suppressalis* larvae that had undergone RCH conditions were re-warmed at 1 °C/min to 25 °C and maintained at the temperature for 0, 0.5, 1, and 2 h prior to exposure to the discriminating temperature for 2 h. Then, the larvae were re-warmed again at 1 °C/min to 25 °C and thereafter transferred individually to glass tubes with artificial diet and assessed for survival as above. The tests were replicated 10 times.

### 2.6. Statistical Analysis

Survival data were subjected to one-way analysis of variance (ANOVA), where applicable, for low temperature treatment effects and means were separated by Tukey HSD test. A two-way ANOVA was used to detect the extent of RCH response, where survival rate is the responding variable and RCH treatment and the sub-zero temperature are the two predicting variables. The data were arcsine-square root transformed and tested for homogeneity of variance before ANOVA. All the statistical analyses were performed using SPSS 13.0 for Windows (IBM, Armonk, NY, USA).

## 3. Results

### 3.1. Discriminating Temperature

**Figure 1:**
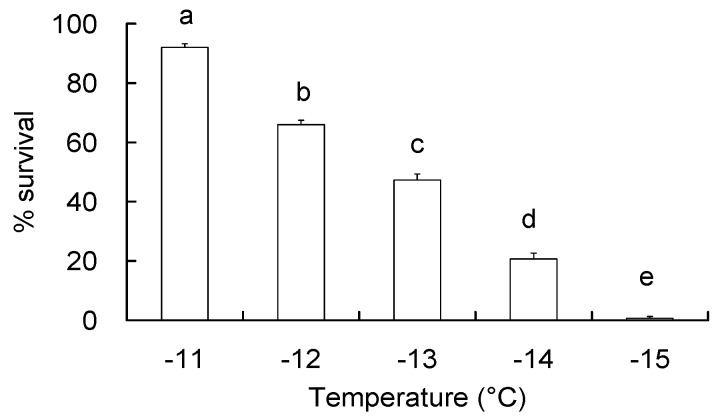
Survival (means ± SE) of diapausing *C. suppressalis* larvae for determination of discriminating temperature. The larvae were directly transferred to sub-zero temperatures from rearing temperature (25 °C) and maintained there for 2 h and then examined 24 h after the larvae were re-warmed to 25 °C. Different letters over the bars indicate significant differences (Tukey HSD test, *p* < 0.05).

To determine the discriminating temperature that results in 80% mortality, diapausing *C. suppressalis* larvae were directly exposed from 25 °C to sub-zero temperatures for 2 h. Their mean survival is shown in Figure 1. Survival of the larvae ranged from nearly zero to more than 90% and was significantly reduced (*F* = 361.55, df = 4,45, *p* < 0.001) with each 1 °C reduction in temperature (Tukey HSD, *p* < 0.001). Larvae exposed to −11, −12, −13, −14, and −15 °C survived at 92%, 66%, 47%, 21%, and less than 1%, respectively. Therefore, the discriminating temperature that resulted in approximately 80% mortality following 2 h exposure was determined to be −14 °C.

### 3.2. RCH Response

**Figure 2:**
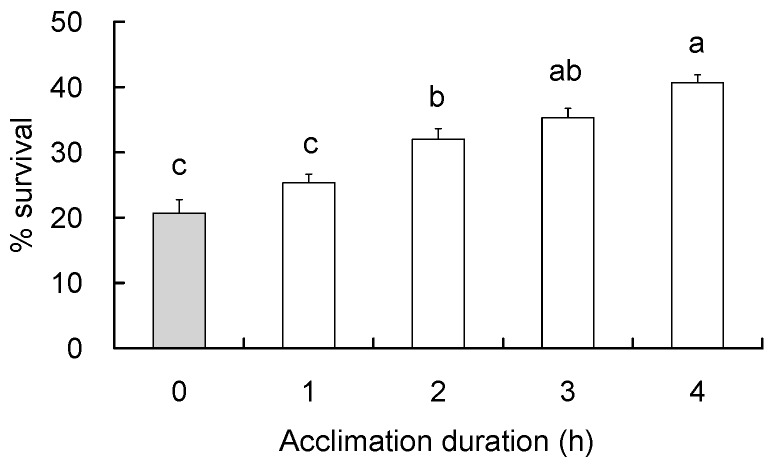
Survival (means ± SE) of diapausing *C. suppressalis* larvae in detection of RCH response. The larvae were exposed to 5 °C for 0–4 h and then transferred to the discriminating temperature (−14 °C) and maintained there for 2 h. Survival was examined 24 h after the larvae were re-warmed to 25 °C from −14 °C. Different letters over the bars indicate significant difference (Tukey HSD test, *p* < 0.05).

The diapausing *C. suppressalis* larvae were subjected to 5 °C for 0–4 h and then immediately to the discriminating temperature for 2 h to detect if RCH occurs in the diapausing larvae. After the treatment, the larvae survived at significantly different rates (*F* = 24.62, df = 4,49, *p* < 0.001; Figure 2). Survival of the rapidly cold-hardened (i.e., 1–4 h at 5 °C) larvae was enhanced by 4.7–20.0%. The RCH effect on survival became significant with 2-h acclimation at 5 °C (an increase of 11% over no RCH, i.e., 0 h at 5 °C) and reached even higher with acclimation for 4 h (an increase of 20 percentage points over no RCH).

### 3.3. Extent of RCH Response

**Figure 3:**
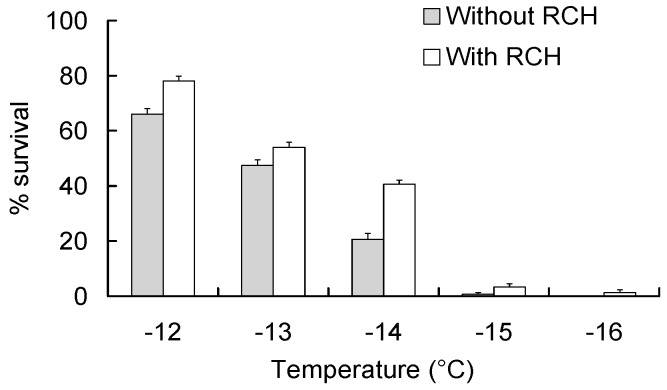
Survival (means ± SE) of diapausing *C. suppressalis* larvae at sub-zero temperatures for effects of RCH. The larvae either rapidly cold hardened at 5 °C for 4 h or not cold-hardened were transferred to the sub-zero temperatures and maintained for 2 h. Survival was examined 24 h after the larvae were re-warmed to 25 °C from the sub-zero temperatures.

To examine the extent of RCH response, the diapausing *C. suppressalis* larvae that had either experienced RCH at 5 °C for 4 h or not were subjected to sub-zero temperatures (−12, −13, −14, −15, and −16 °C) in 10 groups of 15 larvae. The larvae that experienced no RCH did not survive at all at −16 °C and at −15 °C, only one out of 15 larvae survived in just two among the 10 groups (Figure 3). The RCH-experienced larvae survived only one out of 15 at −16 °C and at −15 °C in just two and five among the ten groups, respectively. Due to the near zero survival at both −15 and −16 °C, the survival data at these temperatures were not included in the final statistical analysis. At the other three temperatures, survival of the larvae was significantly influenced by the sub-zero temperature (*F* = 249.49, df = 3,60, *p* < 0.001), the RCH (*F* = 79.07, df = 2,60, *p* < 0.001), and their interaction (*F* = 8.00, df = 2,60, *p* = 0.001; Figure 3). Survival rates were reduced with each 1 °C reduction in temperature (Tukey HSD, *p* < 0.001) and were higher in the cold-hardened larvae than in the larvae that experienced no RCH. 

### 3.4. Durability of RCH Response

**Figure 4:**
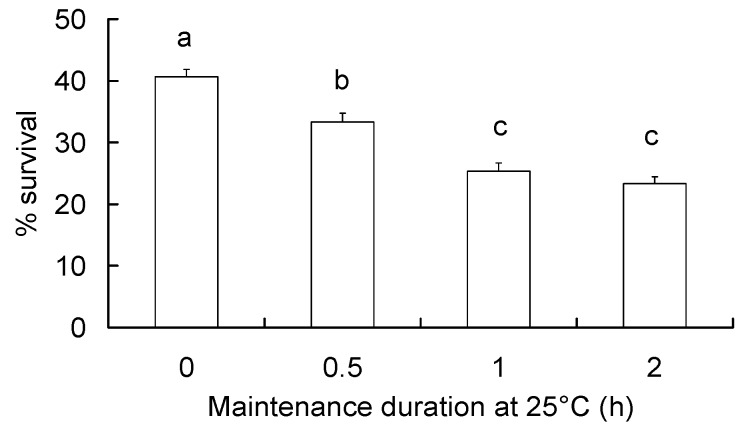
Survival (means ± SE) of diapausing *C. suppressalis* larvae in tests for durability of RCH effect. The RCH-experienced larvae were transferred directly or after maintenance of 0.5, 1 or 2 h at 25 °C, to the discriminating temperature (−14 °C) and maintained there for 2 h. Survival was examined 24 h after the larvae were re-warmed to 25 °C from −14 °C. Different letters over the bars indicate significant difference (Tukey HSD test, *p* < 0.05).

Durability of RCH response was tested through maintaining the RCH-experienced diapausing larvae (acclimated at 5 °C for 4 h) for different durations at the rearing temperature (25 °C) before they were exposed to the discriminating temperature. With these treatments, their survival differed significantly (*F* = 37.97, df = 3,39, *p* < 0.001; Figure 4). The survival rates were significantly decreased by 8% (Tukey HSD, *p* = 0.002), 16% (Tukey HSD, *p* < 0.001) and 18% (Tukey HSD, *p* < 0.001) for the 0.5, 1, and 2 h maintenance durations, respectively, as compared to that without maintenance at 25 °C. Further, the larvae that experienced RCH and post-RCH maintenance were compared to the larvae directly transferred to the discriminating temperature (i.e., no RCH). It was found that the larvae differed significantly in their survival rates (*F* = 28.63, df = 4,49, *p* < 0.001). However, the larvae that experienced RCH and were maintained for 1 and 2 h post RCH showed similar survival rates with the larvae that did not experience RCH (Tukey HSD, *p* > 0.125). 

## 4. Discussion

Different temperature acclimation regimes can induce RCH in terrestrial arthropods. In the present study, RCH was induced in diapausing *C. suppressalis* larvae after a short exposure (2–4 h) at 5 °C. In many other insects, RCH has been induced at temperatures ranging 0–5 °C [7,8,21,22,23,24,25], or even at 10 °C for the predatory mite *Euseius (Amblyseius) finlandicus* [21] and the fruit fly *Bactrocera oleae* [22] and −2 °C for a Karoo beetle *Afrinus* sp [26]. In non-diapausing *C. suppressalis* mature larvae, a previous report showed that RCH could be induced at 0 °C for 4 h and to a lesser extent, at 5 °C for 4 h [10]. Apart from the abrupt cooling (insects are transferred directly from rearing temperature to a lower acclimation temperature), gradual cooling at rates of 0.5 °C per minute or lower has been documented to induce RCH in several insect species [3,8,13,21,22,27]. Whether RCH can be induced in diapausing *C. suppressalis* larvae after a short exposure to gradual cooling has to be further examined.

In several insect species, induction of RCH could be accomplished in as short as 0.5 h at temperatures between 0 and 5 °C [7]. In the present study, 2 h exposure to 5 °C resulted in a significant increase (by 11%) of survival in diapausing *C. suppressalis* larvae exposed to the discriminating temperature and an increase of 20% was recorded with a 4 h exposure. This contrasts to the non-diapausing *C. suppressalis* mature larvae [10], where a 2 h exposure to 5 °C was not able to induce RCH. Similarly, the acclimation durations for a considerable increase in the effects of cold hardening are 1–2 h in *Euseius finlandicus* [21] and *Bactrocera oleae* [22], which are also freeze tolerant species. 

The protection from cold shock gained through RCH for the diapausing *C. suppressalis* larvae was lost within 1 h of their return to 25 °C, decreasing by 18 percentage points. In the rapid-cold hardened non-diapausing *C. suppressalis* mature larvae, survival gained through RCH decreased from more than 45% to around 20% when the RCH-experienced larvae were maintained for 1 h at room temperature before their exposure to the discriminating temperature [10]. The same situation occurred in *Musca domestica* [3], *F. occidentalis* [8], *Psacothea hilaris* [9], and *Corythucha ciliate* [23], while in *E. finlandicus* [21], it lasted for 2 h and for 0.25 h in *B. oleae* [22]. These results indicate that the cold tolerance gained through RCH is plastic [21]. However, in contrast to the results of Qiang et al. [10], our results show that the protection gained through RCH was lost at a lower rate in the diapausing *C. suppressalis* larvae compared to non-diapausing larvae. Moreover, in the natural environment, the diapausing larvae may experience abrupt drops of temperature in the autumn and early spring. However, an immediate rapid increase in temperature is rare, as that administered in the tests for the durability of RCH response. It can be reasoned that the protection gained through RCH may not be lost so quickly in the natural environment as in this study. Therefore, RCH may play a role in preparing the diapausing overwintering *C. suppressalis* larvae for abrupt temperature drops in the field that would otherwise be lethal.

The mechanisms underlying RCH responses have not been extensively explored. In a recent review, Teets, N.M. and Denlinger, D.L. [12] summarized that canonical cold-hardening mechanisms are either not associated with RCH or evidence for their involvement in RCH is inconclusive. The RCH-related physiological adjustments are often small [12]. RCH only elicited few changes in the abundance of detectable proteins in *D. melanogaster* [13]; and in *Locusta migratoria*, heat shock proteins were generally down-regulated [24]. According to Teets, N.M. and Denlinger, D.L. [12], cryoprotectant synthesis, membrane restructuring, adjustments to ion transport mechanisms, and cell-mediated signalling pathways are potential mechanisms associated with RCH. In contrast, the up-regulation of stress-related genes and synthesis of anti-freeze proteins and/or ice nucleating agents are not likely connected with RCH. Further studies of the mechanisms underlying RCH and the relation between RCH effects and SCP change in this species will be performed. 

## 5. Conclusions

Our results indicate that RCH can be induced in diapausing *C. suppressalis* larvae through a short time exposure (2–4 h) to a low temperature of 5 °C, and this confers increased survival at sub-zero temperatures. Although RCH effects on sub-zero temperature survival is lost within 1 h on return of the RCH-experienced diapausing larvae to the room temperature, the protection gained through RCH is relatively strong in the diapausing larvae compared with non-diapausing larvae of *C. suppressalis*. Thus, it is reasonable to assume that RCH that may occur in diapausing *C. suppressalis* larvae during the late autumn and early winter may contribute to its overwintering success.
